# Efficacy of exercise interventions for women during and after gynaecological cancer treatment – a systematic scoping review

**DOI:** 10.1007/s00520-023-07790-8

**Published:** 2023-05-17

**Authors:** Grace Laura Rose, Elizabeth Mary Stewart, Briana Kristine Clifford, Tom George Bailey, Alexandra Jane Rush, Claudia Rose Abbott, Sandra Christine Hayes, Andreas Obermair, Alexandra Leigh McCarthy

**Affiliations:** 1 School of Human Movement and Nutrition Sciences, The University of Queensland, Brisbane, Australia; 2 School of Health, University of the Sunshine Coast, Sippy Downs, Australia; 3 School of Nursing, Midwifery and Social Work, The University of Queensland, Brisbane, Australia; 4 School of Health Sciences, University of New South Wales, Sydney, Australia; 5 School of Health Sciences and Social Work, Griffith University, Brisbane, Australia; 6 Menzies Health Institute Queensland, Griffith University, Brisbane, Australia; 7 School of Medicine, The University of Queensland, Brisbane, Australia; 8 Queensland Centre for Gynaecological Cancer, Royal Brisbane and Women’s Hospital, Brisbane, Australia; 9 Mater Research Institute, Brisbane, Australia

**Keywords:** fitness, strength, body composition, oncology, agility

## Abstract

**Purpose:**

To systematically synthesise evidence of exercise intervention efficacy for physical/psychosocial outcomes that matter to women during/following treatment for gynaecological cancer.

**Methods:**

Five databases were searched (PubMed, EMBASE, CINAHL, PsychInfo, Scopus). Exercise-only intervention studies that included women during/ following treatment for any gynaecological cancer, with/ without control comparison, on any physical or psychosocial outcome(s), were included and qualitatively appraised using the Revised Cochrane Risk of Bias tool and a modified Newcastle-Ottawa Scale.

**Results:**

Seven randomised controlled trials (RCTs), three single-arm pre-post studies, and one prospective cohort study satisfied were included (11 studies). Most studies were completed following treatment (91%), included combined (aerobic and resistance; 36%) and aerobic (36%) training, were fully/mostly (63%) unsupervised, and had a moderate-to-high risk of bias. Overall, 33 outcomes (64% objectively-measured) were assessed. Improvements were observed in aerobic capacity (V̇O_2_ Peak +1.6 mL/kg/min, 6-minute walk distance +20-27 m), lower- (30-second sit-to-stand +2-4 repetitions) and upper-limb strength (30-second arm curl +5 repetitions; 1RM grip strength/chest press +2.4-3.1 kg), and agility (timed up-and-go -0.6 seconds). However, changes in quality of life, anthropometry/body composition, balance and flexibility were inconsistent. There was no evidence to support worsening of outcomes.

**Conclusion:**

Preliminary research into the role of exercise post-gynaecological cancer suggests an improvement in exercise capacity, muscular strength, and agility which, in the absence of exercise, typically decline following gynaecological cancer. Future exercise trials involving larger and more diverse gynaecological cancer samples will improve understanding of the potential and magnitude of effect of guideline-recommended exercise on outcomes that matter to patients.

**Supplementary Information:**

The online version contains supplementary material available at 10.1007/s00520-023-07790-8.

## Introduction

Originating in the female reproductive system, gynaecological cancers encompass malignancies of the ovaries, uterus, cervix, vagina, vulva and fallopian tubes [1]. The incidence of these cancers is rising, with 1,398,601 women diagnosed worldwide in 2020 - a 28% increase in the past 12 years [2, 3]. Whilst gynaecological cancers are often grouped together, they are in fact diverse, with differences in survival [4, 5], recurrence, and treatment, that typically involves varied combinations of surgery, chemotherapy, and radiation therapy [4–6]. This drives differences in recovery and side-effects from treatment, including lower limb lymphoedema, fatigue, depression, anxiety symptoms, fibrosis, pain, and reduced physical function (including strength and muscle mass), fitness/ stamina, and quality of life (QoL) across gynaecological cancer types and individuals [7–12], and requires tailored intervention.

Regular ‘doses’ of aerobic and resistance exercise have acute, pluripotent effects across many bodily systems (e.g., vascular, endocrine, immune, muscular, lymphatic) that amount to system improvement with consistent exercise training [13–17]. This makes tailored exercise an ideal intervention to improve many health-related side effects and symptoms of cancer and its treatment. Position statements from national [18, 19] and international [20] exercise oncology guidelines advocate for regular aerobic and strength training for people with cancer. The evidence underpinning these recommendations predominantly considers improved physical and psychological outcomes for people with breast, prostate, and colorectal cancers, with some evidence embracing gynaecological cancer available for health-related QoL and anxiety [21]. The relative scarcity of evidence in gynaecological cancer was highlighted by a systematic review and meta-analysis in 2016 that examined interventions that included exercise for women with gynaecological cancer, yielding only seven randomised controlled trials over five patient groups (*n*=221) [22]. It was concluded that exercise intervention leads to improvements in physical activity (PA) and body mass index, but no change in fatigue, depression and health-related QoL, and there were insufficient data to determine effect on muscle strength, functional exercise capacity and sexual function [22]. As such, improving health-related outcomes for the increasing number of women who have received a diagnosis of gynaecological cancer is of great clinical importance across all treatment phases and diagnoses.

Remaining gaps in the gynaecological cancer and exercise literature include an understanding of the influence of exercise on health-related physical and psychosocial outcomes, especially considering interventions that isolate the effect of exercise alone (that is, without intervention components additional to exercise) [22]. In the past five years the evidence base to understand these gaps has grown. With a larger base of research now available, it will be possible to include exercise-only studies, therefore reducing the potential influence of other factors on outcomes of interest, whilst also synthesising newly available literature. Therefore, the aim of this systematic review is to evaluate and synthesise the available evidence examining the efficacy of exercise interventions in women with gynaecological cancer on all available health outcomes of interest, essential to recovery during and post-treatment for cancer.

## Materials and methods

### Search strategy

This systematic review was conducted in accordance with the Preferred Reporting Items for Systematic reviews and Meta Analyses (PRISMA) statement [23], and was registered with Prospero (ID: CRD42021222740). Searches were conducted up to 18/03/2022, including databases: PubMed, EMBASE, CINAHL, PsychInfo, and Scopus. Our search included combinations of the free-text terms amalgamated with Boolean operators: (gynecolog*, gynaecolog*, female genitalia, uter*, endometrial, cervix, cervical cancer, ovar*, vagin*, vulva*, fallopian tube*, placenta*, genital*) AND (cancer, neoplasm, carcinoma) AND (Exercise, Physical Therapy Modalities, Exercise Therapy, physical activit*, rehab*, physical therap*, physiotherap*, exercise*, physical function) NOT (animal, mice, rat).

### Selection criteria

The inclusion criteria were: (i) design: RCT, quasi-RCT, cohort, or pre-post single arm studies; (ii) population: women ≥18 years with a diagnosis of gynaecological cancer including malignant tumour of the ovaries, uterus, cervix, vagina, vulva, and fallopian tubes (*a priori* minimum threshold for study inclusion >80% of participants with gynaecological cancer, excluding borderline tumours, or studies that presented results separately for women with gynaecological cancer); (iii) intervention: exercise training (pre-determined block of prescribed physical exercise sessions), including PA (unplanned or unstructured movement/ movement recommendations), physical therapy or exercise monitoring (self- or objective-reporting of exercise of PA over a set time frame); (iv) control: usual care or comparison group receiving an intervention of lesser intensity and/or duration or no comparison group, respectively; (v) any physical (e.g., cardiorespiratory fitness, muscular strength, body composition, physical function, PA) or psychosocial (e.g., QoL, stress, sleep, mental health, sexual function scores) health-related outcomes that are known to be affected by cancer and its treatment, measured through subjective and/or objective methods. The exclusion criteria included publications that were unable to determine the isolated effects of exercise [i.e., those that included multimodal intervention (e.g., combined exercise and diet intervention), complete decongestive therapy (CDT), pelvic floor exercises or dilator use], language other than English, full text not available, or animal study.

### Article appraisal and data extraction

To exclude articles outside the scope of the review, all titles and abstracts were screened by two independent reviewers, including E.S, A.R, C.A and C.K. Following screening and automatic and manual duplicate exclusion, full text articles were independently reviewed by E.S, A.R, C.A, C.K, G.R and B.C against the inclusion and exclusion criteria. Discrepancies were resolved by discussion and a third arbiter was included as necessary. The reference lists of included articles were searched for additional eligible studies, and corresponding authors of eligible studies were contacted in the instance that full text was not available or further information was required. Data extraction were completed using pre-defined data extraction spreadsheet and included details regarding authors; publication year; country; study design (e.g., allocation, blinding); participant characteristics (e.g., age, cancer type and stage); intervention characteristics (e.g., intensity, frequency); sample size; attendance and adherence; and objective (e.g., cardiorespiratory fitness) and subjective (e.g., QoL) outcome measure results. Data were extracted by four reviewers (A.R, E.S, G.R and B.C) and cross-checked by E.S and G.R with any conflicts resolved through discussion. Quantitative (mean difference and statistical significance reported in the study for group post-mean comparison or pre-post intervention comparison depending on the trial design), descriptive, and narrative tools were used to analyse and synthesise the results in table form.

### Methodological quality assessment

The quality of included articles was independently assessed by four authors (A.R, E.S, G.R and B.C), with consensus reached through discussion and final decision by E.S or G.R. All included RCTs were appraised using the Revised Cochrane Risk of Bias tool for randomised trials [24] while a modified Newcastle-Ottawa Scale (MOD-NOS) for non-controlled studies was used as detailed by Heywood, et. al. (2018) [25]. The Cochrane tool for RCTs examines five domains relating to the design (i.e., randomisation, allocation concealment), conduct (i.e.., awareness of assigned intervention, appropriate analysis), outcome data (i.e., missing outcome data), measures (measure appropriateness, knowledge of intervention/ grouping bias), and analysis (i.e., pre-specified analysis plan, multiple eligible results to report) that could cause the effect of an intervention to be favoured toward one group. Each category is rated as having either a low, some concerns, or a high level of bias and contributes to an overall risk of bias rating. As per the MOD-NOS, non-controlled studies were appraised for risk of selection bias and other potential confounding factors. For this review, the MOD-NOS scale focussed on one main study domain of evaluation, which was broken up into subcategories. The scale uses rankings from 0 to 3 points to ascertain risk of bias (0, high risk; 1, mostly high risk; 2, mostly low risk; 3, low risk). A higher overall score is indicative of higher methodological quality. The quality assessment did not influence inclusion of studies; all identified studies were included in the review regardless of rating.

## Results

### Search and selection of studies

**Fig. 1:**
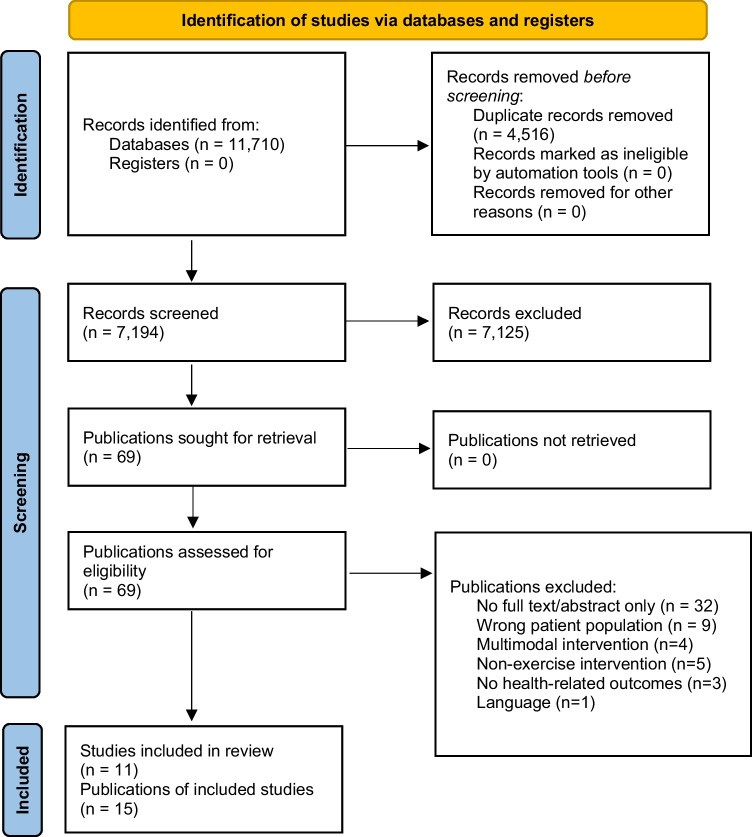
PRIMSA diagram

As seen in Fig. 1, initial database searches produced a total of 11,710 publications from which 4,516 duplicates were removed. Title and abstract screening were completed on 7,194 articles resulting in 69 publications which proceeded to full-text screening. A total of 11 studies reported across 15 publications met inclusion criteria.

### Study design and quality assessment

Of the 11 included studies, seven were RCTs (two with a randomised, waitlist control design) [26–34], three were single-arm pre-post studies [35–39], and one was a prospective cohort study with a historical control [40]. Of controlled trials, most were compared to usual care alone (n=4) or usual care with additional weekly phone contact (n=2). Other studies compared to an information book and weekly phone contact (n=1) or a non-exercising control (n=1).

Overall, most randomised controlled and non-controlled studies included in this review entailed a moderate risk of bias, except for two RCTs which had a high risk of bias [26, 34] (ESM1 and ESM2). For RCTs, the risk of bias assessment item with the best rating (lowest risk of bias) was selection of the reported results. The items with poorest rating (highest risk of bias) included randomisation process, and measurement of the outcome (ESM1). For non-controlled studies, the items with the lowest risk of bias included the use of an appropriate outcome measure and reporting of outcome methodology, and objective assessment of the primary outcome. The items with the highest risk of bias included adequate sample size, identification and adjustment of confounders, and cohort representative of the population of interest (ESM2).

### Participant characteristics

Participant characteristics across the 11 studies are summarised in Table 1. The 11 studies included 640 participants (range n=12-144), with 407 (range n=6-100) allocated to intervention groups. Four studies included ovarian cancer only, three included endometrial cancer only, and four included a combination of endometrial, ovarian, cervical, uterine, vulva, peritoneal and mixed gynaecological. Considering studies collectively, most participants had a diagnosis of ovarian cancer (52%), followed by endometrial cancer (34%). The proportion of women with other listed cancer diagnoses was between <1% for vulva cancer and 6% for cervical cancer. On average, participants were aged 57 years (range=51-64 years; Table 1), and most were categorised as being obese according to BMI (average=30 kg/m^2^; range=24.3-40.1 kg/m^2^; Table 1). Disease stage at diagnosis varied, with most participants diagnosed with Stage I disease (44%), followed by Stage III (29%), Stage II (15%) and Stage IV (12%; Table 1); however, some studies (n=3) did not report cancer stage. The predominant treatment reported was chemotherapy (57%), where other treatments included surgery (37%) and radiotherapy (16%). Most participants had only one type of treatment, and interventions (91%) were conducted post-treatment except for one study where participants were on active treatment [39]. Participants were approximately two years post-diagnosis at time of participation (n=8 studies; range 4-46 months).

**Table 1:** Participant characteristics of included studies

Study Citation Country	Sample size Total *n* Group *n*	Retention rate %	Diagnosis cancer type/s (%)	Months since diagnosis mean±SD (range)	Cancer stage Stage (%)	Treatment Type (%)	Age mean±SD	BMI mean±SD
1a. Armbruster et al., 2016 [35] USA	**100**	63%	Endometrial (100%)	27.6±15.6	I (71%) II-III (29%)	Surgery (56%) Surgery + Radio (33%) Surgery + Radio + Chemo (11%)	58±10	34.0±9.7
1b. Basen-engquist et al., 2014 [36] USA	**100**	79%	Endometrial (100%)	26.6±15.2	I (80%) II (16%) III (4%)	Surgery (58%) Surgery + Radio (42%)	57±11	34.2±9.4
1c. Robertson et al., 2019 [37] USA	**100**	74%	Endometrial (100%)	26.6±15.2	I (80%) II (16%) III (4%)	Surgery (58%) Surgery + Radio (42%)	57±11	34.2±9.4
2. Cartmel et al., 2021 [26] USA	**144** I 74 C 70	78%	Ovarian (100%)	20.2±12.0	I (24%) II (21%) III (40%) IV (15%) NR (1%)	Chemo (93%) Unknown (7%)	I 57±9 C 57±9	I 29.0±7.2 C 29.1±6.8
3a. Crawford et al., 2016 [27] Canada	**35** I 24 C 11	97%	Endometrial (40%) Ovarian (31%) Cervical (29%)	NR	NR	NR	I 53±13 C 54±11	I 26.1±5.1 C 27.3±5.0
3b. Crawford et al., 2017 [28] Canada	**35** I 24 C 11	97%	Endometrial (40%) Ovarian (31%) Cervical (29%)	NR	NR	NR	I 53±13 C 54±11	I 26.1±5.1 C 27.3±5.0
4. Donnelly et al., 2011 [29] Ireland	**33** I 16 C 17	I 75% C 100%	Endometrial (34%) Ovarian (36%) Cervical (12%) Uterine (12%) Gynaecological (6%)	8.7±9.1	I (49%) II (30%) III (21%)	Chemo (39%) Radio (21%) Chemo + Radio (39%)	I 54±9 C 52±12	I 29.6±8.3 C 30.0±7.8
5. Gorzelitz et al., 2022 [30] USA	**40** I 20 C 20	95%	Endometrial (100%)	34.8±15.0	I (83%) II (5%) III (12%)	Surgery (75%) Chemo (8%) Radio (7%) Chemo + Radio (10%)	I 61±10 C 61±8	I 42.2±19.5 C 37.9±8.6
6. Hausmann et al., 2018 [31] Norway	**60** I 21 C 16	90%	Ovarian (25%) Cervical (27%) Uterine (47%) Vulva (1%)	NR	NR	Surgery (93%) ^1^ Chemo (47%) ^1^ Radio (13%) ^1^	57±13	NR
7a. Iyer et al., 2018 [32] USA	**95** I 50 C 45	I 84% C 82%	Ovarian (100%)	18.8±10.8	I (27%) II (23%) III (32%) IV (18%)	Surgery (76%) ^1^ Chemo (88%) ^1^	I 58±9 C 59±7	I 28.8±7.1 C 29.9±6.6
7b. Zhou et al., 2017 [33] USA	**144** I 74 C 70	I 82% C 74%	Ovarian (100%)	20.4±12.0	I (24%) II (21%) III (40%) IV (15%)	Chemo (93%) NR (7%)	I 57±9 C 57±9	I 29.0±7.2 C 29.1±6.8
8. Lee et al., 2021 [40] South Korea	**12** I 6 C 6	NR	Ovarian (100%)	46.2±6.8	NR	Surgery (100%)	I 50±10 C 52±6	I 24.6±2.4 C 24.0±4.1
9. Mizrahi et al., 2016 [38] Australia	**30**	70%	Ovarian (100%)	During Tx	I/II (20%) III/IV (80%)	Chemo (100%)	59±11	25.3±7.1
10. Newton et al., 2011 [39] Australia	**17**	100%	Ovarian (76%) Peritoneal (24%)	NR	I (6%) II (6%) III (64%) IV (24%)	Chemo (100%)	60±8	<18 (n=2), 18-<25 (n=4), ≥25-<30 (n=2), ≥30 (n=2), NR (n=7)
11. Rossi et al., 2016 [34] USA	**28** I 13 C 15	68%	Endometrial (100%)	32.0±19.0	I (86%) III (11%) IV (3%)	Chemo + Radio (24%) NR (76%)	64±8	36.6±6.5

Bolded numbers correspond to total sample size of the study

*BMI* body mass index, *C* control, *I* intervention, *n* number of participants, *NA* not applicable/not measured), *NR* not reported, *SD* standard deviation

^1^ distribution of participants who had multiple treatment types not specified

### Intervention characteristics

The exercise intervention duration evaluated in 11 studies ranged between eight to 24 weeks, with an average of 15 weeks (Table 2), except one study that was completed during treatment that based intervention length on duration of treatment [39]. Most interventions (n=7) were completed at home [26, 29, 30, 32, 33, 35–39], two were completed at a gym only [27, 28, 31], one was completed at a gym and at home [34], and one did not report the location of the intervention [40] (ESM3). Of the 11 interventions, most implemented combined aerobic and resistance training alone [29] or in combination with flexibility [31], balance training [38] or dance [34]. Four interventions included aerobic training alone [26, 32, 33, 35–37, 39], two included resistance training alone [30, 40], and one included indoor rock climbing [27, 28]. Session frequency and duration was reported for 10 interventions and was 3 days per week on average (range 2-5 days/week), with duration of 10 to 120 minutes. All studies had a weekly prescribed exercise volume accumulation of between 90 to 240 minutes (average=167 minutes; Table 2). Aerobic interventions were reportedly completed at a moderate intensity on average, and included mainly walking activities, but also swimming and cycling [38], and dance [34]. Many studies did not report the method or criteria of aerobic intensity measurement [26, 31, 34, 39], but those that did (n=4) measured mostly by rating of perceived exertion (Borg RPE 12-16/20), percentage of age-predicted heart rate maximum (55-70% APHRM) and heart rate reserve (40-90% HRR; ESM3). Interventions that involved resistance training were reportedly prescribed at a moderate to vigorous intensity overall, by one-repetition maximum (50-70% 1RM), RPE (Borg RPE 11-14/20, 7-8/10 OMNI-res), and repetition maximum (6-12 RM). Whole body, body weight, free weight and resistance banded exercise of 10-12 repetitions and two to three sets were prescribed. No studies reported progression of the interventions.

**Table 2:** Summary of intervention characteristics of the included studies

	Aerobic only (*n*=4)	Resistance only (*n*=2)	Combined (*n*=4)	Other (*n*=1)
Mode	Walking	Upper and lower body, machine, free weight, Theraband resistance exercises; individual prescription	Walking, cycling, swimming, aerobic dance; whole body and core stability resistance training with free and machine weights, both individual and group classes; balance; flexibility	Indoor wall climbing
Intensity	Low to high	Moderate to vigorous	Moderate to high	Not reported
Session duration	30-45 minutes per session performed as continuous aerobic exercise; 150/minutes per week	55-60 minutes per session	25-90 minutes per session	120 minutes per session
Frequency	If reported, 4-5 days per week	2-4 days per week	2-5 days per week	2 days per week
Intervention duration	18-24 weeks	10-12 weeks	12-16 weeks	8 weeks
Progression	Not reported	Not reported	Not reported	Not reported
Cancer type	Ovarian (69%), Endometrial (25%), Other (6%)	Endometrial (50%), Ovarian (50%)	Endometrial (48%), Ovarian (40%), Cervical (10%), Other (2%)	Endometrial (40%), Ovarian (31%), Cervical (29%)

Supervision, where a qualified health professional attended and watched exercise sessions via face-to-face or video, was reported in 10 studies. Of these studies, 40% were fully unsupervised, 30% were mostly unsupervised, 10% were mostly supervised, and 20% were fully supervised. Studies that were fully unsupervised provided additional support through regular, weekly telephone support [26, 29, 30, 32, 33, 35–37], a face-to-face familiarisation session at commencement [30], and/or provision of educational materials [32, 33] (ESM3). Exercise attendance was reported in four studies and was 89% on average. Exercise adherence to at least one component of the intervention was reported in six studies, and was 69% for aerobic components, 75% for resistance components and 123% as related to achieving 150 minutes of activity per week (ESM3). Retention rate to RCTs was 90% on average (range=78-100%), and 78% on average for non-controlled studies (range=68-100%; Table 1). In general, the RCT and single arm/ cohort studies did not greatly differ in method or location of delivery, support services, participant retention or exercise prescription.

### Objective and physical outcomes

The objective outcomes reported across studies included aerobic capacity/fitness (n=8, 73%), muscular strength (n=6, 55%), body composition (n=7, 64%), physical function (n=3, 27%) and PA (n=3, 27%; Table 3). The most common method of assessment for each outcome included the 6-minute walk test (6MWT) distance (n=4), 30-second chair stand (n=4), waist circumference (n=4), sit and reach, back scratch and timed up and go (n=2), and 7-day PA recall (n=2; ESM4).

**Table 3:** Summary of intervention outcomes of the included studies

Outcomes	n^**1**^	Levels of evidence^1^			Cancer type^1^				n^2^	Statistically significant results^2^	
		II	III	IV	Endo	Cerv	Ovar	Oth		Improvement	No change
Exercise capacity	5	2 (40%)	2 (40%)	1 (20%)	57%	8%	29%	6%	5	3 (60%)	2 (40%)
Cardiorespiratory fitness	3	1 (33%)		2 (67%)	49%	9%	42%		3	1 (33%)	2 (66%)
Upper limb strength	5	2 (40%)	1 (20%)	2 (40%)	37%	11%	51%	1%	8	6 (75%)	2 (25%)
Lower limb strength	5	2 (40%)	2 (40%)	1 (20%)	57%	31%	11%	1%	7	5 (71%)	2 (29%)
Body mass	3	2 (67%)	1 (33%)		62%	22%	14%	2%	3		3 (100%)
Central adiposity	4	2 (50%)	1 (25%)	1 (25%)	72%	17%	10%	3%	4		4 (100%)
Body composition	2		1 (50%)	1 (50%)	50%		50%		3		3 (100%)
LLL	1	1 (100%)					100%		1		1 (100%)
Agility	2	2 (100%)			70%	15%	15%		2	2 (100%)	
Balance	1			1 (100%)			100%		1	1 (100%)	
Flexibility	2	2 (100%)			70%	15%	15%		4	1 (25%)	3 (75%)
Physical activity	3	1 (33%)		2 (67%)	49%	4%	45%	2%	3	2 (67%)	1 (33%)
HRQoL	8	3 (38%)	2 (25%)	3 (37%)	48%	5%	43%	4%	15^3^	7 (47%)	8 (53%)
Fatigue	1	1 (100%)			46%	12%	36%	6%	1	1 (100%)	
Stress	1			1 (100%)	100%				1		1 (100%)
Mental wellbeing	2	1 (50%)		1 (50%)	50%		50%		2	1 (50%)	1 (50%)
Sleep	1			1 (100%)	100%				1		1 (100%)

^1^Numbers and percentages relative to studies reporting each outcome

^2^Numbers and percentages relative to all outcomes measured in each study; difference either between groups or over time, depending on trial design

^3^Mental and physical component scores for instruments (e.g., SF-36) considered as separate outcomes

*Cerv*: cervical cancer, *Endo*: endometrial cancer, *HRQoL*: health-related quality of life, *LLL*: lower limb lymphoedema, *Oth*: other gynaecological cancers, *Ovar*: ovarian

#### Aerobic exercise capacity and cardiorespiratory fitness

Five RCTs assessed aerobic capacity/fitness, where three studies demonstrated a significant between-group difference in favour of the exercise group for both V̇O_2_ Peak (+1.6 mL/kg/min) and 6MWT distance (+20-27 metres) at the end of intervention [28, 31, 34] (Table 3; ESM4). However, two studies did not demonstrate a significant effect of their intervention on aerobic capacity but did report an average improvement of 20 metres and 52 metres. These improvements were similar to significant improvements seen in other studies, as assessed by 6MWT and 12-minute walk test, respectively [29, 30]. Pre-post studies were equivocal, in that two studies did not improve aerobic capacity [36, 38] and one study did [39].

#### Muscular strength

Four RCTs assessed lower limb muscular strength. The three studies that assessed lower limb strength via the 30-second chair stand reported significant between-group differences post-intervention favouring the exercise groups (range: +2-4 repetitions) [28, 30, 34] (Table 3; ESM4). For other measures of lower limb strength among RCTs, isotonic knee extensor strength and leg press did not improve when compared between groups [31]. However, significant improvements in lower limb strength (leg press) were noted in a study was assessed pre- compared to post-study [38]. Overall, five of seven outcomes reported across five studies demonstrated a positive effect of exercise on lower limb strength, with level II-IV evidence.

Upper limb strength was assessed in three RCTs, where studies reported significant improvements compared to control in a 30-second arm curl test in two studies (+5 repetitions) [28, 30], but not during a 1RM chest press in one study (+2.4 kg) [31] (Table 3; ESM4). There was equivocal evidence for improvement in grip strength, where one study reported significant improvement compared to control (+3.1 kg) [28], and one did not (+0.5 kg) [30]. A prospective cohort study also reported significant between-group improvement compared to control (10.6 kg) [40]. For other assessments of upper limb and core strength, single-arm and prospective cohort studies reported improvement in 10RM seated row performance (+3.4 kg) [38] and 1-minute sit-up test performance (+12 repetitions) [40]. Overall, six of eight upper limb strength outcomes improved in five studies of level II-IV evidence.

#### Body composition

Six RCTs assessed body composition and reported no influence of exercise on waist circumference [28, 29, 34], body mass [28, 34], BMI [29], body fat percentage or lean mass [30] when compared to control (Table 3). One study that employed a waitlist-controlled design found an improvement in the exercise group over time (12 weeks) favouring reduction in waist circumference (-5.4 cm), however this effect was isolated [34] (ESM4). There did not appear to be an influence of exercise training on gynaecological cancer lymphoedema outcomes when assessed by lower limb lymphoedema prevalence [32] compared to control within an RCT. Within a study of lower-level evidence (prospective cohort study) there was an influence of exercise on change over time (12 weeks) for improvements lean mass (+5.8 kg) [40], but not compared to control.

#### Physical function

Two RCTs reported improvement in agility assessed by 8-foot up-and-go (average -0.6 sec) [28, 30]. Studies reported no change in upper limb flexibility assessed by the back scratch test, and mixed evidence for improvement in posterior chain flexibility, with a significant improvement in one study (-1.1 cm) and no change in another for the sit and reach test [28, 30] (Table 3; ESM4). One single-arm study assessed balance through a single leg balance assessment and reported significant pre- compared to post-intervention improvement following the exercise intervention [38].

#### Physical activity (PA)

One RCT assessed the difference in self-reported PA (7-day recall) in intervention compared to control, and found that PA did not increase in the intervention group [29]. Two single-arm studies that assessed exercise by accelerometry and self-report questionnaires found a significant improvement in PA minutes per day (average +4 mins/day) [36] and MET-hours per week (8 MET-hours/week) [38], respectively (Table 3; ESM4).

### Self-reported outcomes

The most common subjective measure was QoL (n=11 studies, n=15 composite scores, 92%), which was most consistently assessed via the Short-form 36 (SF-36) across pooled studies (n=5; Table 3). Other outcome measures for the assessment of QoL included general (n=1), endometrial (n=2) and ovarian-specific (n=2) Functional Assessment of Cancer Therapy (FACT), Quality of Life in Adult Cancer Survivors (QLACS, n=2), and Patient-reported Outcomes Measurement Information System (PROMIS, n=1) instruments. Further subjective outcomes included fatigue, (n=1), stress (n=1), mental wellbeing (n=2), and sleep quality (n=1; Table 3; ESM5).

#### Quality of life (QoL)

Of the five RCTs that measured QoL, three studies (60%) reported significant improvements in global QoL/composite scores (*p*=0.02-0.05) compared to control [27, 33, 34] (Table 3; ESM5). These studies also demonstrated significant improvements compared to control in sub-scales, including physical function [33], social functioning [33], endometrial-specific QoL [34] and general health [33] (ESM5). Within controlled trials, there were several sub-scales that did not demonstrate any significant effect of exercise compared to control, including: fatigue, cognitive functioning, pain, mental component scores/mental health, vitality, and role limitations.

Of the three single-arm studies that reported QoL, two reported significant improvements in global QoL or component summaries (*p*=0.001-0.027) when compared to pre-intervention [35, 38] (Table 3). This was consistent with results seen among controlled trials. Following the intervention there were significant improvements in sexual function- and interest-specific mental scores, sexual problems, negative/positive feelings, cognitive problems, pain, fatigue, and social avoidance [35] (ESM5). Similar to controlled trials, there were also significant improvements noted in the sub-domains of physical function and physical component scores [36, 38], along with general health [36] and functional wellbeing [38, 39], but no notable improvement in vitality or role limitations. Unlike controlled trials, there were significant improvements in a mental component score [38], bodily pain [36] and ovarian-specific QoL [39], but no significant improvement in social wellbeing. Overall, seven of 15 outcomes reported across eight studies demonstrated a positive effect of exercise on QoL, with level II-IV evidence.

#### Fatigue

One RCT assessed the influence of exercise training on fatigue in isolation, and reported that exercise training significantly improved (reduced) fatigue, relative to control (*p*=0.046) [29] (Table 3). However, it was notable that there was not improvement in fatigue in QoL subscales, as aforementioned.

#### Stress

One single-arm study reported the influence of exercise training on stress and found no influence of exercise on global stress score (*p*=0.792) [36]. This outcome was not investigated in isolation across any other study (Table 3).

#### Mental wellbeing

In the two randomised controlled trials that assessed mental wellbeing, exercise training was shown to improve anxiety (*p*<0.05), depression (*p*<0.05) in one study [30] and in the other study (*p*=0.05; Table 3) [26]. The inconsistent effect of exercise on mental wellbeing was further identified in a single-arm study, where exercise did not affect anxiety and depression following training but did improve the tendency to somatization (*p*=0.03) [36].

#### Sleep

One single-arm study reported the influence of exercise training on global sleep score and found no influence of exercise following the intervention (*p*=0.831; Table 3) [37].

## Discussion

Despite substantial heterogeneity in study designs and participant characteristics, the greatest and most consistent effect of exercise was seen in the improvement of muscular strength, exercise capacity, and agility, all of which are known to be reduced in response to cancer and its treatment among women [41, 42]. These improvements in turn potentially reduce fatigue [43, 44] and improve an individual’s ability to maintain activities of daily living for enhanced QoL [43]. Importantly, these outcomes are supported by mostly level II evidence, and both exercise capacity and lower limb strength were two of the most assessed physical outcomes, yielding a greater amount of pooled data than other objective outcomes. There did not appear to be an influence of exercise on body fat and anthropometry change, and there was insufficient evidence for changes to flexibility, balance, stress, sleep, mental wellbeing, and fatigue. There were mixed effects for change to QoL, despite a comparable number of outcomes and level of evidence to outcomes that were most consistently improved (i.e., exercise capacity, lower limb strength, agility). These results indicate that exercise could have some potential benefit on some but not all health-related outcomes known to contribute to increased likelihood of incidence and QoL due to physical and psychological influences [42, 45, 46].

Many of the included interventions were powered to assess feasibility, acceptability and safety of the intervention, and cited sample size as a study limitation for the analysis of effect (the outcomes of interest for this review) [28, 31, 34, 38, 39]. Overall, median sample size for included studies was 35 (range: 12-144), which may rationalise why a consistent intervention effect was not seen among studies. Further, as physical outcomes were often not the priority of studies to-date, studies are limited in the comprehensiveness of physical and psychosocial outcome assessment. Many outcomes of interest, particularly psychosocial and body composition outcomes, are prone to ceiling effects (attenuation when scores that are at or near to the possible upper limit) and require large sample sizes. For example, QoL outcomes such as the SF-36 tend to have small, but potentially clinically significant changes in their global scores among men and women [47], and typically require a large sample size to detect change. Sample size calculations or estimates of statistical power were only conducted in two studies [30, 40], so it is not possible to conclude whether the other studies were sufficiently powered to detect statistical changes in primary and secondary subjective and objective outcomes. It is pertinent that future studies include sample sizes that are appropriate to quantify the effect of exercise interventions - notwithstanding the notorious difficulty in the recruitment and retention of participants in exercise trials.

Despite numerous studies that have found collective improvements in QoL and body composition among other cancer groups (e.g., breast and prostate) [33, 48–51] similar effects were not seen in this review. Assessment techniques used to assess physical and psychosocial outcomes to date have varied in quality, which concurrently may influence the collective evidence of this systematic review. For example, the SF-36 is more reactive to clinical than statistical improvements and is considered more psychometrically sound than tools such as QLACS [52–54]. Furthermore, it is well known that the assessment of body composition is affected by techniques used, where DXA and compartment models are most appropriate to assess change over time [55]. Within the present study, 73% of studies that included subjective/ psychosocial outcomes (QoL) and 33% of studies that included objective/physical outcomes (aerobic capacity, muscular strength, body composition, PA) utilised composite and psychometrically-sound questionnaires and reference (gold-standard) techniques, respectively (SF-36, FACT, VO2 peak, 1RM, DXA, accelerometry) [47, 55–59]. Whilst QoL outcomes reported in this review are likely valid, there remains a clear need for future research that implements gold-standard assessment techniques in women during and after treatment for gynaecological cancer, especially for physical outcomes.

Given the heterogeneity of study types, sample sizes and prescriptions, as well as the lack of differentiation between gynaecological tumours, it is not possible to determine the influence of different cancer types and exercise prescriptions on outcomes. Some inferences, however, can be made. Although the influence of exercise volume, duration and intensity was unclear, exercise type seemed to influence results. Studies that included combined training [29, 31, 34, 38] seemed to have the most consistent influence of exercise training across multiple outcome measures (exercise capacity, muscular strength, fatigue, QoL, balance, PA, body composition) [31, 34, 38]. Although this influence cannot be confirmed in this review, this observation aligns with the current recommendation to embed combined training for people with cancer, within national [18, 19] and international [20] PA guidelines. This is likely because combined exercise training (including components of aerobic and resistance training) improves multifaceted physical outcomes, such as cardiorespiratory fitness and strength, that cannot be targeted by one modality alone. Multiple training modalities maximise exercise adaptations simultaneously as per current exercise guidelines for people with cancer [18–20], which might lead to greater improvements in QoL and other outcomes linked to self-efficacy (e.g., PA). Studies to-date do not yet include sufficient sample size to understand participant characteristics that pre-dispose them to being responders/ non-responders to exercise training (e.g., cancer type, stage, treatment). As exercise and gynaecological cancer studies progress in sample size and assessment of efficacy outcomes, particularly understanding how different gynaecological cancer types respond to exercise will become an important avenue for future research.

Although safety and feasibility was not systematically reviewed here, pooled findings from included efficacy studies reinforce that exercise during and after treatment is likely to be feasible. All of the included studies that assessed feasibility determined the programs were feasible (n=6), as assessed by recruitment rate, PA adherence and focus group evaluation [29], retention [34, 38], 76% rate of completion of chemotherapy dose [39], or reported in text without specification [26, 33]. Within the present review, it was also clear that the type of exercise and level of supervision played an important role in the studies that did assess intervention acceptability. Included studies suggested that intervention acceptability was most positive with regular calls and consultations based upon qualitative feedback [29]; that lack of social support and less self-discipline present as barriers to exercise [38]; and the impact on QoL appears to be stronger with involvement in more social forms of PA [37]. Despite apparent feasibility, we have limited capacity to draw conclusions about the safety of interventions. Only four interventions (36%) reported on adverse events, but of these interventions, none reported any adverse events other than muscle soreness that could be attributed to the exercise intervention [32, 34, 38, 39]. The participants across the eleven studies were diagnosed across a range of cancer stages from I to IV and underwent various treatments (e.g., surgery, hormone therapy). The lack of reported adverse events, though incomplete, appears to indicate that exercise is safe for the variety of women during and after treatment for gynaecological cancer. Despite inconsistent results across all outcomes, exercise in gynaecological cancer populations appears to be feasible and safe, based on the limited evidence to date.

There are several limitations to this review. The median sample sizes of the collective studies were relatively small (median = 35); indeed, many of the studies included were pilot RCTs that were not sufficiently powered to detect statistical changes in secondary study outcomes. Given the generally small sample sizes, it was not appropriate to sub-analyse the results based on disease stage, treatment type or cancer population, which limits our ability to establish how generalisable these results are to the many women treated for gynaecological cancer. Although the inclusion of single-arm studies was required to comprehensively assess the existing evidence for the influence of exercise on physical and psychosocial outcomes among women during and following treatment for gynaecological cancer, the limited inclusion of RCTs (64%) reduces certainty in the outcomes reported here, as does the low-moderate risk of bias of included studies. Furthermore, an insufficient number of studies and variability in outcomes assessed and methods of assessment meant that participants could not be analysed collectively via meta-analysis. Given the promising outcomes seen here, future studies must now extend this pilot work by recruiting larger samples and, where possible, the use of gold-standard techniques to quantify health-related outcomes. Future rigorous and consistent assessment practice will ultimately inform robust clinical exercise practice. Finally, reported intervention characteristics were limited, which diminished our ability to assess integral components of the interventions such as attendance, compliance, supervision, progression, and modalities of exercise prescribed.

Overall, the results from this systematic review support the inclusion of exercise training, including low-moderate-vigorous gym, group, or home-based aerobic and strength exercise among women with gynaecological cancer, for the benefit of aerobic capacity, muscular strength, and agility, with level II-IV evidence. There might be a more pronounced benefit of combined exercise training on physical and psychosocial outcomes. Whilst this review presents the best collective evidence to date regarding the influence of exercise on health-related outcomes following gynaecological cancer treatment, future research should include studies that are appropriately powered and focus on the inclusion of gold-standard techniques for the assessment of physical and psychosocial outcomes, which is presently limited. It remains unknown whether combined exercise that aligns with the current international PA recommendations for people living with and beyond varied gynaecological cancer is sufficient to improve patient-centred psychosocial outcomes (especially QoL). This must be investigated in larger participant samples in studies that are of high quality (level II evidence, low risk of bias).

Exercise is shown here to improve health-related outcomes that are known to decline in response to cancer and its treatment. Notwithstanding important future directions, exercise, including whichever prescription is best tolerated and preferred, should be implemented among women with a diagnosis of gynaecological cancer, especially ovarian and endometrial cancers who hold the majority of the evidence.

## Supplementary information

ESM 1 (DOCX 45 kb)

ESM 2 (DOCX 46 kb)

ESM 3 (DOCX 72 kb)

ESM 4 (DOCX 99 kb)

ESM 5 (DOCX 98 kb)
