# Supplementary material for: Efficacy of exercise interventions for women during and after gynaecological cancer treatment – a systematic scoping review
Source: Support Care Cancer. 2023 May 17;31(6):342. doi: 10.1007/s00520-023-07790-8 (PMC10191940; doi:10.1007/s00520-023-07790-8)
Supplement: Supplementary file 1 — (DOCX 45 kb) [file 520_2023_7790_MOESM1_ESM.docx]

**Table A.1.** Revised Cochrane Risk of Bias Tool summary for randomised controlled trials

|  | Randomization process ^a^ | Deviations from the intended assignment to intervention ^b^ | Deviations from the intended adherence to intervention ^c^ | Missing outcome data ^d^ | Measurement of the outcome ^e^ | Selection of the reported result ^f^ | Overall bias |
| --- | --- | --- | --- | --- | --- | --- | --- |
| 2. Cartmel et al. 2021 | High | Low | Some concerns | Low | Low | Low | High |
| 3. Crawford et al. 2016^g^ | Low | Low | Low | Low | Some concerns | Low | Some concerns |
| 4. Donnelly et al. 2018 | Low | Low | Some concerns | Low | Low | Low | Some concerns |
| 5. Gorzelitz et al. 2022 | Low | Some concerns | Low | Some concerns | Low | Low | Some concerns |
| 6. Hausmann et al. 2018 | Some concerns | Low | Low | Some concerns | Low | Low | Some concerns |
| 7. Zhou et al. 2017^g^ | Low | Low | Low | Low | Some concerns | Low | Some concerns |
| 11. Rossi et al. 2016 | High | High | Some concerns | Some concerns | Some concerns | Low | High |

Assessment of randomised, controlled trials, using the Revised Cochrane Risk of Bias Tool (ROB-2), as Low: low risk of bias, Some concerns: moderate risk of bias, High: high risk of bias

^a^ Bias arising from randomization process

^b^ Bias due to deviations from the intended interventions (effect of assignment to intervention)

^c^ Bias due to deviations from the intended interventions (effect of adhering to intervention)

^d^ Bias due to missing outcome data

^e^ Bias in measurement of the outcome

^f^ Bias in selection of the reported result

^g^ For trials where multiple publication of the same study were used, the earliest publication was used to determine the risk of bias for the study as a whole
