# Supplementary material for: Efficacy of exercise interventions for women during and after gynaecological cancer treatment – a systematic scoping review
Source: Support Care Cancer. 2023 May 17;31(6):342. doi: 10.1007/s00520-023-07790-8 (PMC10191940; doi:10.1007/s00520-023-07790-8)
Supplement: Supplementary file 2 — (DOCX 46 kb) [file 520_2023_7790_MOESM2_ESM.docx]

**Table A.2.** Modified Newcastle-Ottowa Scale summary for non-controlled and cohort studies

|  | Selection bias | Performance bias | | Detection bias | | Information bias | | Total risk |
| --- | --- | --- | --- | --- | --- | --- | --- | --- |
|  | Is the source population appropriate and representative of the population of interest? | Is the sample size adequate to detect a meaningful difference in the outcome of interest? | Did the study identify and adjust for any variable or confounders that may influence the outcome? | Did the study use appropriate statistical analysis methods relative to the outcomes of interest? | Is there little missing data and did the study handle it accordingly? | Is the methodology of the outcome measurement explicitly stated and is it appropriate? | Is there an objective assessment of the outcome of interest? |  |
| 1. Basen-engquist et al. 2014^a^ | 1 | 1 | 2 | 2 | 3 | 3 | 3 | 15 (Mod) |
| 8. Lee et al. 2021 | 2 | 1 | 1 | 2 | 1 | 3 | 3 | 13 (Mod) |
| 9. Mizrahi et al. 2016 | 1 | 1 | 2 | 2 | 2 | 3 | 3 | 14 (Mod) |
| 10. Newton et al. 2011 | 2 | 1 | 1 | 2 | 3 | 3 | 3 | 15 (Mod) |

Assessment of non-controlled/ cohort studies, using the Modified Newcastle-Ottowa Scale (MOD-NOS), using the following categories for each question – 0/ definitely no: high risk of bias, 1/ mostly no: mostly high risk of bias, 2/ mostly yes: mostly low risk of bias, 3/ definitely yes: low risk of bias. A total risk score of 16-21 was classified as low risk, 8-15 as moderate risk, and 0-7 as high risk of bias.

^a^ For trials where multiple publication of the same study were used, the earliest publication was used to determine the risk of bias for the study as a whole
