# Supplementary material for: Efficacy of exercise interventions for women during and after gynaecological cancer treatment – a systematic scoping review
Source: Support Care Cancer. 2023 May 17;31(6):342. doi: 10.1007/s00520-023-07790-8 (PMC10191940; doi:10.1007/s00520-023-07790-8)
Supplement: Supplementary file 3 — (DOCX 72 kb) [file 520_2023_7790_MOESM3_ESM.docx]

**Table A.3.** Study and intervention characteristics of the included studies

| **Citation**  Country  Design | **Study details**  Duration (D; weeks)  Location (L)  Supervision (S)  Control (C) | **Exercise Prescription Details**  Frequency (F; days per week)  Intensity (I; classification, RPE/ HR%/ 1RM%)  Time (T; minutes per session)  Mode (M) | **Adherence**  Attendance (A; %)  Compliance (C; %) | **Outcomes Assessed**  Outcome (test/ technique) |
| --- | --- | --- | --- | --- |
| **1a. Armbruster et al., 2016 [30]**  USA  Single-arm pre-post | D: 24 wks  L: Home  S: Unsupervised + weekly calls  C: NA | F: ≥ 5 days/wk  I: Mod., RPE 12-16/20 (BORG)  T: 30 min  M: AEx (Walking) | A: NR  C: NR | Sexual int. + func. (SF-36, QLACS) |
| **1b. Basen-Engquist et al., 2014** [31]  USA  Single-arm pre-post | D: 24 wks  L: Home  S: Unsupervised + weekly calls  C: NA | F: ≥5 days/wk  I: Mod., RPE 12-16/20 (BORG)  T: 30 min  M: AEx (Walking) | A: NR  C: NR | QoL (SF-36, QLACS, PSS, BSI-18)  CRF (V̇O_2_ Peak)  Body comp (WC)  PA (mins/day) |
| **1c. Robertson et al., 2019 [32]**  USA  Single-arm pre-post | D: 24 wks  L: Home  S: Unsupervised  C: NA | F: ≥5 days/wk  I: Mod., RPE 12-16/20 (BORG)  T: 30 min  M: AEx (Walking) | A: NR  C: NR | QoL (PSQI) |
| **2. Cartmel et al., 2021 [21]**  USA  RCT | D: 24 wks  L: Home  S: Unsupervised + weekly calls  C: Usual care + weekly calls | F: NR  I: Mod., HR Rx NR  T: 150 mins/wk  M: AEx (mainly brisk walking) | A: I=82%, C=86%^1^  C: 115%^1^ | Depression (CED-S) |
| **3a. Crawford et al., 2016 [22]**  Canada  Pilot RCT | D: 8 wks  L: Climbing gym  S: NR  C: Usual care | F: 2 days/wk  I: NR  T: 120 min  M: Indoor wall climbing | A: 84%  C: NR | QoL (SF-36) |
| **3b. Crawford et al., 2017 [23]**  Canada  Pilot RCT | D: 8 wks  L: Climbing gym  S: NR  C: Usual care | F: 2 days/wk  I: NR  T: 120 min  M: Indoor wall climbing | A: 84%  C: NR | CRF (6MWT)  Musc. strength (30 STS, arm curl, GS)  Power (TUG)  Body comp (WC, body mass)  Flexibility (S&R + BS) |
| **4. Donnelly et al., 2011 [24]**  Ireland RCT | D: 12 wks  L: Home  S: Unsupervised + weekly calls  C: Usual care + weekly calls | F: ≥5 days/wk  I: Mod., 12-13/20 (BORG)  T: 30 min  M: AEx (walking), RT (Rx NR) | A: NR  C: 58% (completed >60 mins AEx) | QoL (FACT-G)  Fatigue (MFSI-SF)  CRF (12MWT)  Body comp (BMI, WC)  PA (mins/wk) |
| **5. Gorzelitz et al., 2022 [25]**  USA  Pilot RCT | D: 10 wks  L: Home  S: Unsupervised + 1 FTF familiarisation Sx + weekly calls  C: Usual care | F: 2 days/wk  I: Mod.-vig., 7-8/10 (OMNI-res)  T: ~60 min  M: RT (12 reps, 2-3 sets) w/ TB + DBs | A: 96% (wks 1-10), 99% (wks 11-15)  C: >75% | QoL (FACT-En, PROMIS)  CRF (6MWT)  Musc. strength (30 STS, arm curl, GS)  Power (TUG)  Body comp (BF%, LM; DXA)  Flexibility (S&R + BS) |
| **6. Hausmann et al., 2018 [26]**  Norway  RCT | D: 16 wks  L: Gym (group)  S: Supervised  C: No exercise | F: 2 days/wk  I: Mod-high, Ax method NR  T: 90 min (WU 15 min, AEx 30 min. RT 25 min, flexibility + relax 20 min)  M: AEx, RT and flexibility (Rx NR) | A: NR  C: NR | CRF (V̇O_2_ Peak)  Musc. strength (leg press, extension, chest press) |
| **7a. Iyer et al., 2018 [27]**  USA  RCT | D: 24 wks  L: Home  S: Unsupervised + info book + weekly calls  C: Info book + weekly calls | F: NR  I: Mod., 40-90% HRR  T: 150min/wk  M: AEx (walking) | A: NR  C: NR | LD (prevalence) |
| **7b. Zhou et al., 2017 [28]**  USA  RCT | D: 24 wks  L: Home  S: Unsupervised  C: Info book + weekly calls | F: NR  I: Mod., 40-90% HRR  T: 150min/wk  M: AEx (walking) | A: I=87%, C=82%^1^  C: 110%^1^ | QoL (SF-36) |
| **8. Lee et al., 2021 [35]**  South Korea  Prospective cohort study | D: 12 wks  L: NR  S: Supervised  C: Usual care | F: 4 days/wk  I: Mod.-vig., 12 RM (wk 1-3), 10RM (wk 4-6), 8RM (wk 7-9), 6RM (wk 10-12)  T: 50-60 min  M: RT (details NR) | A: NR  C: NR | Musc. strength (GS)  Musc. endurance (1 min sit-up)  Body comp (LM; BIS) |
| **9. Mizrahi et al., 2016 [33]**  Australia  Single-arm pre-post | D: 12 wks  L: Home  S: 75% unsupervised, 25% supervised  C: NA | F: 3-4 days/wk  I: Low-mod., RPE 11-14/20, AEx 55-70% HRmax; RT 50%-70% 1RM  T:10-40 min, 90 min/wk  M: AEx (walking, cycling swimming), RT (whole body + core stability, 10 reps, 3 sets), balance (NR) | A: NR  C: 71% achieved 90 mins PA 75% of study | QoL (FACT-O, SF-36)  CRF (V̇O_2_ Peak)  Musc. strength (30STS, leg press, seated row)  Balance (single leg)  PA (MET hours/wk) |
| **10. Newton et al., 2011 [34]**  Australia  Single-arm pre-post | D: Length of Tx (chemo; ~18 weeks)  L: Home  S: ~75% unsupervised, ~25% supervised  C: NA | F: ≥4 days/wk  I: Mod-high, Ax method NR  T: 20-60 min  M: AEx (Walking) | A: 90%  C: >80% supervised, 76% unsupervised | QoL (FACT-O)  CRF (6MWT) |
| **11. Rossi et al., 2016 [29]**  USA  Wait-list controlled trial | D: 12 wks  L: Gym and home  S: 57% supervised (dance + RT), 43% unsupervised (walking)  C: Usual care | F: 2 days/wk  I: Mod.-vig., Ax method NR  T: 60min (Sx; 5 min WU, 25 min dance, 20 min RT, 10 min CD) + 90min/wk (walking)  M: Aerobic dance, RT (Rx NR), walking | A: 86% (to Sx)  C: 130% (for walking time), Sx NR | QoL (FACT-En)  CRF (6MWT)  Musc. strength (30STS)  Body comp (WC, body mass) |

6MWT: 6 minute walk test, 12MWT: 12-minute walk test, 30STS: 30-seconds sit to stand test, AEx: aerobic exercise, APHRM: age predicted heart rate maximum, BF%: body fat percentage, BIS: bioelectrical impedance spectroscopy, BMI: body mass index, BS: back scratch, BSI-18: Brief symptom inventory 18, C: control, CD: cool down, CDT: complete decongestive therapy, CED-S: centre for epidemiologic studies depression scale, CRF: cardiorespiratory fitness, D: duration, DB: dumbbells, DXA: dual-energy x-ray absorptiometry, EORTC QLQ-C30: European organisation for research and treatment of cancer core quality of life questionnaire, F: frequency, FACT: functional assessment of cancer therapy, FTF: face to face, func.: function, GS: grip strength, HR: heart rate, HRmax: heart rate maximum, I: intensity, int.: interest, LD: lymphoedema, L: location, LL: lower limb, LM: lean mass, M: modality, min: minutes, mod: moderate, musc.: muscular, NR: not reported, PA: physical activity, PROMIS: patient-reported outcomes measurement information system, PSQI: Pittsburgh sleep quality index, PSS: perceived stress scale, QoL: quality of life, QLACS: quality of life in adult cancer survivors, RCT: randomised controlled trial, reps: repetitions, RPE: rating of perceived exertion, RT: resistance training, Rx: prescription, S: supervision, SF-36: short form 36, S&R: sit and reach, Sx: session, T: time, TB: theraband, TUG: timed up and go, Tx: treatment, UL: upper limb, USA: United States of America, vig.: vigorous, WC: waist circumference, wks: weeks, WU: warm up, w/: with

^1^ Attendance: attending weekly phone calls, Compliance: as related to achieving 150 mins of aerobic activity per week
