# Supplementary material for: Efficacy of exercise interventions for women during and after gynaecological cancer treatment – a systematic scoping review
Source: Support Care Cancer. 2023 May 17;31(6):342. doi: 10.1007/s00520-023-07790-8 (PMC10191940; doi:10.1007/s00520-023-07790-8)
Supplement: Supplementary file 4 — (DOCX 99 kb) [file 520_2023_7790_MOESM4_ESM.docx]

**Table A.4.** Results of objective and physical outcomes of the included studies

| Citation  Country  Design | Objective outcome measure | n | Intervention | | n | Control | | *p* group, time |
| --- | --- | --- | --- | --- | --- | --- | --- | --- |
|  |  |  | **BL mean±SD (range)** | **Post mean±SD (range)** |  | **BL mean±SD (range)** | **Post mean±SD (range)** |  |
| *Aerobic Exercise Capacity* | | | | | | | | |
| 1b. Basen-Engquist et al., 2014 [31]  USA  Single-arm pre-post | V̇O_2_ Peak (mL/kg/min) | 100 | *OB:*17.0±5.4  *N-OB:*21.8±8.4 | *OB:*18.3±4.5  *N-OB:* 22.3±5.3 | - | -  - | -  - | NA, 0.143 |
| 6. Hausmann et al., 2018 [26]  Norway  RCT | V̇O_2_ Peak (mL/kg/min) | 29 | 29.7±8.0 | 31.3±8.3 | 26 | 28.1±6.3 | 28.2±7.5 | 0.037*, 0.009^ |
| 9. Mizrahi et al., 2016 [33]  Australia  Single-arm pre-post | V̇O_2_ Peak (mL/kg/min) | 21 | 24.4±5.9 | 24.9±6.8 | - | - | - | NA, 0.500 |
| 3b. Crawford et al., 2017 [23]  Canada  RCT | 6MWT (m) | 24 | 564.0±70.0 | 591.0±60.0 | 11 | 554.0±72.0 | 538.0±77.0 | <0.001^, NR |
| 5. Gorzelitz et al., 2022 [25]  USA  Wait-list controlled trial | 6MWT (m) | 20 | 472.1±96.9 | 492.3±109.1 | 20 | 478.8±69.5 | 477.9±10.6.4 | 0.290, NR |
| 10. Newton et al., 2011 [34]  Australia  Single-arm pre-post | 6MWT (m) | 17 | 332.0 (266.0-256.0) | 395.0 (356.0-460.0) | - | - | - | NA, 0.010^ |
| 11. Rossi et al., 2016 [29]  USA  Wait-list controlled trial | 6MWT (m) | 17 | 431.0±64.0 | 453.0±65.0 | 12 | 427.0±60.0 | 428.0±65.0 | <0.010^, <0.010 |
| 4. Donnelly et al., 2011 [24]  Ireland  RCT | 12MWT (m) | 16 | 873.0±176.4 | 925.6±175.9 | 17 | 888.0±150.0 | 928.7±184.6 | 0.760, NR |
| *Muscular Strength* | | | | | | | | |
| 3b. Crawford et al., 2017 [23]  Canada  RCT | 30sec Chair Stand (reps)  30sec Arm Curl (reps)  Grip Strength Right (kg)  Grip Strength Left (kg) | 24 | 17±4  19±4  31.8±5.5  30.2±5 | 21±5  23±4  34.9±5.2  32.7±5.3 | 11 | 18±5  19±4  31.6±6.4  29.7±7 | 18±5  19±5  31.6±6.9  28.9±7.3 | <0.001^, NR  <0.001^, NR  0.013*, NR  0.024*, NR |
| 5. Gorzelitz et al., 2022 [25]  USA  Wait-list controlled trial | 30sec Chair Stand (reps)  30sec Arm Curl (reps)  Grip Strength Dominant (kg) | 20 | 12±3  16±3  22.0±6.0 | 15±5  21±6  22.5±6.4 | 20 | 11±3  14±3  21.8±5.4 | 11±3  17±4  21.2±4.7 | <0.001*, NR  0.001*, NR  0.110, NR |
| 11. Rossi et al., 2016 [29]  USA  Wait-list controlled trial | 30sec Chair Stand (reps) | 17 | 13±3 | 14±3 | 12 | 11±3 | 12±3 | <0.010^, >0.020 |
| 9. Mizrahi et al., 2016 [33]  Australia  Single-arm pre-post | 30sec Chair Stand (reps)  Supine Leg Press 10RM (kg)  Seated Row 10RM (kg) | 21 | 14±5  28.1±7.5  22.4±5.0 | 18±5  35.9±11.8  25.8±5.5 | - | -  -  - | -  -  - | NA, 0.004^  NA, 0.003^  NA, 0.001^ |
| 8. Lee et al., 2021 [35]  South Korea  Prospective cohort study | Grip Strength Dominant (kg)  1 min sit-up (reps) | 6 | 32.6±4.7  6±2 | 43.2±4.3  18±2 | 6 | 32.1±6.9  5±3 | 26.5±7.4  3±3 | <0.010^, <0.050*  <0.010^, <0.050* |
| 6. Hausmann et al., 2018 [26]  Norway  RCT | Chest Press 1RM (kg)  Leg Press 1RM (kg)  Leg Extension 1RM (kg) | 29 | 24.5±7.5  113±27.3  44.2±10.1 | 26.9±8.2  116.7±29.2  48.0±10.6 | 26 | 25.7±8.6  108±35.5  45.8±16.5 | 25±8.7  110.5±31.3  48.3±16.4 | >0.050, 0.001^  >0.050, 0.047*  >0.050, <0.001 |
| *BOdy Composition* | | | | | | | | |
| 1b. Basen-Engquist et al., 2014 [31]  USA  Single-arm pre-post | Waist Circumference (cm) | 100 | *OB:* 110.5±13.1  *N-OB:*81.8±8.5 | *OB:* 112.1±15.9  *N-OB:* 81.4±9.9 | - | -  - | -  - | NA, 0.670 |
| 3b. Crawford et al., 2017 [23]  Canada  RCT | Waist Circumference (cm)  Body mass (kg) | 24 | 90.3±12.8  72.2±14.5 | 89.4±11.8  72.5±14.4 | 11 | 89.7±10.8  73.1±14.5 | 90.7±11.5  73.7±15.6 | 0.170, NR  0.710, NR |
| 4. Donnelly et al., 2011 [24]  Ireland  RCT | Waist Circumference (cm)  Body mass index (kg/m^2^) | 16 | 88.8±17.9  29.6±8.3 | 88.7±16.8  30.0±8.2 | 17 | 89.1±20.2  30.0±7.8 | 90.8±21.4  30.3±7.6 | 0.120, NR  0.940, NR |
| 11. Rossi et al., 2016 [29]  USA  Wait-list controlled trial | Waist Circumference (cm)  Body mass (kg) | 17 | 113.6±12.9  94.0±16.9 | 108.4±11.4  93.4±15.7 | 12 | 111.7±11.6  97.1±20.9 | 114.2±14.4  97.4±21.8 | >0.200, <0.010*  >0.200, >0.200 |
| 5. Gorzelitz et al., 2022 [25]  USA  Wait-list controlled trial | DXA Body fat percentage (%)  DXA Lean mass (kg) | 20 | 51.6±5.0  47.8±8.4 | 50.6±5.2  47.3±7.6 | 20 | 50.4±6.8  47.2±6.9 | 50.0±7.2  46.3±6.4 | 0.350, NR  0.150, NR |
| 8. Lee et al., 2021 [35]  South Korea  Prospective cohort study | BIS Lean mass (kg) | 6 | 32.7±7.9 | 35.4±7.3 | 6 | 33.2±5.9 | 29.6±2.3 | >0.050, <0.050 |
| 7a. Iyer et al., 2018  USA [27]  RCT | LLL Prevalence (%) | 50 | 32.6 | 27.9 | 45 | 37.8 | 35.1 | 0.490, NR |
| *Physical Function* | | | | | | | | |
| 3b. Crawford et al., 2017 [23]  Canada  RCT | Sit and Reach (cm)  Back Scratch (cm)  8-foot up-and-go (sec) | 24 | 9.0±8.7  1.0±6.8  4.4±0.6 | 12.8±9.2  1.8±7.3  4.5±0.9 | 11 | 8.1±8.7  1.0±7.4  4.4±0.6 | 9.2±8.1  1.0±1.1  4.1±0.6 | 0.016*, NR  0.310, NR  0.039*, NR |
| 5. Gorzelitz et al., 2022 [25]  USA  Pilot RCT | Sit and Reach (cm)  Back Scratch (cm)  8-foot up-and-go (sec) | 20 | -3.1±8.9  -20.3±11.7  6.6±1.3 | -1.0±7.9  -17.5±10.1  5.9±1.0 | 20 | 1.8±10.9  -13.0±13.7  6.9±1.4 | 1.0±7.4  -12.5±12.5  6.7±1.2 | 0.600, NR  0.800, NR  0.030*, NR |
| 9. Mizrahi et al., 2016 [33]  Australia  Single-arm pre-post | Single Leg Balance (sec) | 21 | 30.4±28.6 | 47.1±28.6 | - | - | - | NA, 0.003^ |
| *Physical activity* | | | | | | | | |
| 4. Donnelly et al., 2011 [24]  Ireland  RCT | 7 Day PA Recall (mins/wk) | 16 | 660.0±970.5 | 505.0±858.8 | 17 | 390.0±635.0 | 583.5±990.0 | 0.210, NR |
| 1b. Basen-Engquist et al., 2014 [31]  USA  Single-arm pre-post | Accelerometry Physical Activity (mins/day) | 100 | *OB:* 13.6±12.5  *N-OB:* 16.4±9.9 | *OB:* 16.1±11.4  *N-OB:* 23.0±12.6 | - | -  - | -  - | NA, 0.001^ |
| 9. Mizrahi et al., 2016 [33]  Australia  Single-arm pre-post | IPAQ MET-hours (per week) | 21 | 9.8±8.4 | 18.1±10.6 | - | - | - | NA, 0.007^ |

6MWT: 6-minute walk test, 12MWT: 12-minute walk test, BIA: bioelectrical impedance analysis, BIS: bioelectrical impedance spectroscopy, BL: baseline, DXA: dual-energy x-ray absorptiometry, IPAQ: international physical activity questionnaire, LLL: lower limb lymphoedema, NA: not applicable, N-OB: non-obese, NR: not reported, OB: obese, PA: physical activity, Post: end/length of intervention, RCT: randomised controlled trial, SD: standard deviation, USA: United States of America, V̇O_2_ Peak: peak volume of oxygen

^denotes significance at *p*<0.01, *denotes significance at *p*<0.05
