# Supplementary material for: Efficacy of exercise interventions for women during and after gynaecological cancer treatment – a systematic scoping review
Source: Support Care Cancer. 2023 May 17;31(6):342. doi: 10.1007/s00520-023-07790-8 (PMC10191940; doi:10.1007/s00520-023-07790-8)
Supplement: Supplementary file 5 — (DOCX 98 kb) [file 520_2023_7790_MOESM5_ESM.docx]

**Table A.5.** Results of self-reported outcomes of the included studies

| Citation  Country | Subjective outcome measure | n | Intervention | | | n | Control | | | *p* group, time |
| --- | --- | --- | --- | --- | --- | --- | --- | --- | --- | --- |
|  |  |  | **BL mean±SD** | **Post mean±SD** | |  | **BL mean±SD** | **Post mean±SD** | |  |
| *Quality of life* | | | | | | | | | | |
| 1a. Armbruster et al., 2016 [30]  USA  Single-arm pre-post | **SF-36**  PCS SI  MCS SI  PCS SF  MCS SF | 100 | -0.1^#^±NR  -0.4^#^±NR  -0.1^#^±NR  -0.3^#^±NR | | NR  NR  NR  NR | - | -  -  -  - | | -  -  -  - | NA, 0.652  NA, 0.001*  NA, 0.196  NA, 0.001* |
| 1b. Basen-Engquist et al., 2014 [31]  USA  Single-arm pre-post | **SF-36**  Physical functioning  Mental health  Vitality  Role limitations – physical  Bodily pain  General health  Social functioning  Role limitations – emotional | 100 | *OB:* 76.3±24.6  *N-OB:* 90.2±10.9  *OB:* 80.9±12.3  *N-OB:* 81.4±15.3  *OB:* 60.0±20.6  *N-OB:* 64.1±16.8  *OB:* 75.6±37.3  *N-OB:* 82.8±31.4  *OB:* 73.1±20.9  *N-OB:* 78.6±18.4  *OB:* 71.0±20.9  *N-OB:* 84.7±10.5  *OB:* 86.3±19.9  *N-OB:* 90.5±17.9  *OB:* 78.3±34.2  *N-OB:* 85.1±27.6 | | NR  NR  NR  NR  NR  NR  NR  NR  NR  NR  NR  NR  NR  NR  NR  NR | - | -  -  -  -  -  -  -  -  -  -  -  -  -  -  -  - | | -  -  -  -  -  -  -  -  -  -  -  -  -  -  -  - | NA, <0.001*  NA, 0.812  NA, 0.172  NA, 0.263  NA, 0.023*  NA, 0.001*  NA, 0.235  NA, 0.441 |
| 3a. Crawford et al., 2016 [22]  Canada  RCT | **SF-36**  PCS  MCS  Physical functioning  Mental health  Vitality  Role limitations – physical  Bodily pain  General health  Social functioning  Role limitations – emotional | 23 | 51.0±8.1  53.6±6.4  53.4±4.4  53.9±6.4  53.4±8.2  51.7±7.3  50.2±10.3  50.8 ±10.4  52.3±6.9  52.8±6.5 | | 50.8±7.8  51.5±7.5  53.3±4.9  53.3±5.8  50.2±8.5  50.5±9.4  48.4 ±10.6  52.2±8.7  51.4±8.1  50.3±7.8 | 11 | 52.5±9.2  45.9±10.7  51.0±7.9  46.9±8.8  47.8±11.1  47.1±11.4  52.3±12.0  52.7±7.2  51.4±8.4  44.6±12.7 | | 54.9±7.5  41.4±12.4  53.0±5.6  44.9±10.7  47.5±11.7  48.6 ±10.6  53.7±9.9  51.0±9.9  45.9±11.2  41.7±13.2 | 0.038*, NR  0.063, NR  0.150, NR  0.077, NR  0.600, NR  0.063, NR  0.690, NR  0.480, NR  0.190, NR  0.120, NR |
| 7b. Zhou et al., 2017 [28]  USA  RCT | **SF-36**  PCS  MCS  Physical functioning  Mental health  Vitality  Role limitations – physical  Bodily pain  General health  Social functioning  Role limitations – emotional | 74 | 46.3±8.9  47.5±11.3  45.3±9.9  47.1±10.4  48.4±10.9  42.5±10.9  51.8±9.5  46.4±9.7  46.8±9.9  45.7±12.5 | | 48.1±NR  49.1±NR  47.6±NR  48.6±NR  51.0±NR  44.6±NR  51.9±NR  48.0±NR  49.2±NR  47.5±NR | 70 | 45.6±9.2  48.9±11.4  45.8±8.1  49.2±9.5  48.3±10.8  41.9±11.1  51.0±9.9  45.4±8.5  48.2±10.6  45.6±11.5 | | 43.6±NR  49.4±NR  43.8±NR  50.0±NR  48.3±NR  41.5±NR  49.2±NR  43.1±NR  46.8±NR  46.0±NR | 0.020*, NR  0.440, NR  <0.001*, NR  0.560, NR  0.080, NR  0.190, NR  0.270, NR  0.004*, NR  0.020*, NR  0.470, NR |
| 9. Mizrahi et al., 2016 [33]  Australia  Single-arm pre-post | **SF-36**  PCS  MCS | 21 | 41.6±9.8  42.5±11.5 | | 46.3±6.1  47.6±8.6 | - | -  - | | -  - | NA, 0.027*  NA, 0.007* |
| 5. Gorzelitz et al., 2022 [25]  USA  Wait-list controlled trial | **FACT-EN**  Global QoL  Physical wellbeing  Social wellbeing  Functional wellbeing  Emotional wellbeing  Endometrial specific | 20 | 146.0±11.9  24.3±2.3  22.3±3.2  20.4±3.3  21.1±2.7  55.0±7.1 | | 146.0±15.0  24.8±2.9  22.3±4.8  20.2±3.4  20.8±3.8  55.0±7.5 | 20 | 141.0±21.7  24.0±3.4  20.6±6.5  21.6±5.0  20.4±3.3  54.5±7.1 | | 140.0±26.1  24.5±4.5  19.7±7.6  21.0±6.3  20.2±3.4  55.0±7.0 | >0.050, <0.050*  >0.050, <0.050*  >0.050, >0.050  >0.050, <0.050*  >0.050, >0.050  >0.050, <0.050* |
| 11. Rossi et al., 2016 [29]  USA  Wait-list controlled trial | **FACT-EN**  Global QoL  Endometrial specific | 25 | 141.0±14.0  53.0±8.0 | | 151.0±17.0  59.0±8.0 | 15 | 143.0±15.0  53.0±6.0 | | 143.0±12.0  54.0±7.0 | 0.030*, 0.050  0.020*, <0.010* |
| 9. Mizrahi et al., 2016 [33]  Australia  Single-arm pre-post | **FACT-O**  Global QoL  Physical wellbeing  Social wellbeing  Functional wellbeing  Emotional wellbeing  Ovarian specific | 30 | 101.3±22.1  18.1±7.0  20.6±5.4  16.3±6.1  15.2±6.0  31.2±6.3 | | 109.9±21.0  21.6±5.9  19.8±6.2  18.9±4.5  17.1±4.3  32.9±5.2 | - | -  -  -  -  -  - | | -  -  -  -  -  - | 0.017*, 0.004*  0.006*, 0.001*  0.368, 0.610  0.009*, 0.006*  0.067, 0.040*  0.447, 0.210 |
| 10. Newton et al., 2011 [34]  Australia  Single-arm pre-post | **FACT-O**  Global QoL  Physical wellbeing  Social wellbeing  Functional wellbeing  Emotional wellbeing  Ovarian specific | 17 | 109.0±NR  18.0±NR  23.0±NR  19.0±NR  20.0±NR  31.0±NR | | 113.0±NR  23.0±NR  22.0±NR  19.0±NR  21.0±NR  36.0±NR | - | -  -  -  -  -  - | | -  -  -  -  -  - | NA, 0.100  NA, 0.080  NA, 0.110  NA, 0.020*  NA, 0.290  NA, 0.040* |
| 4. Donnelly et al., 2011 [24]  Ireland  RCT | **FACT-G**  Global QoL | 16 | 69.9±15.6 | | 80.2±16.9 | 17 | 73.1±14.5 | | 78.7±19.1 | 0.370, 0.250 |
| 5. Gorzelitz et al., 2022 [25]  USA  Wait-list controlled trial | **PROMIS**  Anxiety  Depression  Fatigue | 20 | 46.6±3.4  46.4±3.4  50.9±3.1 | | 46.4±3.4  44.6±3.9  48.1±2.9 | 20 | 47.5±3.3  49.1±2.9  50.9±3.1 | | 48.9±2.9  48.1±3.4  48.8±3.1 | >0.050, >0.050  >0.050, >0.050  >0.050, >0.050 |
| 1b. Basen-Engquist et al., 2014 [31]  USA  Single-arm pre-post | **QLACS**  Negative feelings  Positive feelings  Cognitive problems  Pain  Sexual problems  Fatigue  Social avoidance  Financial problems  Distress – family  Benefits  Appearance  Distress recurrence | 100 | *OB:* 11.5±4.6  *N-OB:* 11.3±4.2  *OB:* 20.8±4.7  *N-OB:*21.5± 4.4  *OB:* 10.9±4.5  *N-OB:* 11.2±4.3  *OB:* 11.1±5.2  *N-OB:* 8.7±3.4  *OB:* 12.3±6.2  *N-OB:*13.2± 6.5  *OB:* 15.0±3.1  *N-OB:* 14.6±2.7  *OB:* 9.7±5.1  *N-OB:* 8.3±4.3  *OB:* 8.4±6.0  *N-OB:* 6.3±4.3  *OB:* 3.1±1.8  *N-OB:* 2.7±1.5  *OB:* 18.5±6.2  *N-OB:* 17.5±5.6  *OB:* 6.0±2.8  *N-OB: 6.0*±3.1  *OB:* 12.7±6.4  *N-OB:* 9.4±4.1 | | *OB:* 10.0±3.8  *N-OB:* 8.9±4.1  *OB:* 22.4±4.6  *N-OB:*22.7±3.9  *OB:* 9.7±3.8  *N-OB:* 10.0±3.6  *OB:* 9.1±4.6  *N-OB:* 7.6±2.7  *OB:* 9.8±4.7  *N-OB:*11.5±6.0  *OB:* 13.8±2.6  *N-OB:*13.8±3.0  *OB:* 7.8±3.6  *N-OB:* 6.4±3.4  *OB:* 8.0±5.5  *N-OB:* 5.2±1.4  *OB:* 2.7±1.6  *N-OB:* 2.3±1.0  *OB:* 19.1±5.9  *N-OB:*18.8±5.8  *OB:* 5.6±2.5  *N-OB:* 5.3±2.3  *OB:* 10.9±5.8  *N-OB:* 8.1±3.4 | - | -  -  -  -  -  -  -  -  -  -  -  -  -  -  -  -  -  -  -  -  -  -  -  - | | -  -  -  -  -  -  -  -  -  -  -  -  -  -  -  -  -  -  -  -  -  -  -  - | NA, 0.492  NA, 0.877  NA, 0.893  NA, 0.014*  NA, 0.347  NA, 0.543  NA, 0.088  NA, 0.066  NA, 0.323  NA, 0.856  NA, 0.777  NA, 0.013* |
| 1a. Armbruster et al., 2016 [30]  USA  Single-arm pre-post | **QLACS**  Sexual problems SI  Sexual problems SF  Negative feelings SI  Negative feelings SF  Positive feelings SI  Positive feelings SF  Cognitive problems SI  Cognitive problems SF  Pain SI  Pain SF  Fatigue SI  Fatigue SF  Social avoidance SI  Social avoidance SF  Financial problems SI  Financial problems SF  Distress – family SI  Distress – family SF  Benefits SI  Benefits SF  Appearance SI  Appearance SF  Distress recurrence SI  Distress recurrence SF | 100 | 6.8±3.9  4.9±3.1  0.4^#^±NR  0.5^#^±NR  -0.3^#^±NR  -0.3^#^±NR  0.3^#^±NR  0.4^#^±NR  0.3^#^±NR  0.4^#^±NR  0.3^#^±NR  0.4^#^±NR  0.4^#^±NR  0.3^#^±NR  0.2^#^±NR  0.2^#^±NR  0.1^#^±NR  0.2^#^±NR  -0.1^#^±NR  -0.1^#^±NR  0.1^#^±NR  0.1^#^±NR  0.2^#^±NR  0.3^#^±NR | | 6.3±3.8  4.1±2.5  NR  NR  NR  NR  NR  NR  NR  NR  NR  NR  NR  NR  NR  NR  NR  NR  NR  NR  NR  NR  NR  NR | - | -  -  -  -  -  -  -  -  -  -  -  -  -  -  -  -  -  -  -  -  -  -  -  - | | -  -  -  -  -  -  -  -  -  -  -  -  -  -  -  -  -  -  -  -  -  -  -  - | NA, 0.070  NA, 0.002*  NA, <0.001*  NA, <0.001*  NA, 0.006*  NA, 0.003*  NA, 0.005*  NA, <0.001*  NA, 0.001*  NA, <0.001*  NA, 0.004*  NA, <0.001*  NA, <0.001*  NA, 0.004*  NA, 0.100  NA, 0.022*  NA, 0.225  NA, 0.080  NA, 0.363  NA, 0.199  NA, 0.655  NA, 0.336  NA, 0.052  NA, 0.157 |
| *Fatigue* | | | | | | | | | | |
| 4. Donnelly et al., 2011 [24]  Ireland  RCT | **MFSI-SF**  Global score | 16 | 29.6±17.2 | | 9.1±17.1 | 17 | 26.5±22.1 | | 17.5±26.4 | 0.046*, NR |
| *stress* | | | | | | | | | | |
| 1b. Basen-Engquist et al., 2014 [31]  USA  Single-arm pre-post | **PSS**  Global Score | 100 | *OB:* 21.7±7.4  *N-OB:* 23.0±8.1 | | *OB:* 18.90±6.2  *N-OB:*18.20±7.2 | - | -  - | | -  - | NA, 0.792 |
| *mental wellbeinG* | | | | | | | | | | |
| 1b. Basen-Engquist et al., 2014 [31]  USA  Single-arm pre-post | **BSI-18**  Somatization  Anxiety  Depression | 100 | *OB:* 3.3±2.8  *N-OB:* 1.6±2.5  *OB:* 3.5±3.8  *N-OB:* 3.2±2.9  *OB:* 3.7±3.9  *N-OB:* 2.3±3.0 | | *OB:*2.5±2.4  *N-OB:* 1.2±1.3  *OB:* 2.2±2.9  *N-OB:* 2.5±2.4  *OB:*2.7±3.0  *N-OB:* 2.0±3.0 | - | -  -  -  -  -  - | | -  -  -  -  -  - | NA, 0.003*  NA, 0.957  NA, 0.100 |
| 2. Cartmel et al., 2021 [21]  USA  RCT | **CEDS**  Depression | 74 | 23.3±10.1 | | 9.5±8.6 | 70 | 9.2±9.6 | | 9.7±8.9 | 0.050*, 0.01 |
| *SLEEP* | | | | | | | | | | |
| 1c. Robertson et al., 2019 [32]  USA  Single-arm pre-post | **PSQI**  Global score | 100 | -0.693^#^±NR | | NR | - | - | | - | NA, 0.831 |

BL: baseline, BSI-18: Brief symptom inventory 18, CED-S: center for epidemiologic studies depression scale, EORTC QLQ-C30: European organisation for research and treatment of cancer core quality of life questionnaire, FACT-EN: functional assessment of endometrial cancer therapy, FACT-G: functional assessment of general cancer therapy, FACT-O: functional assessment of ovarian cancer therapy, MCS: mental component score, MFSI-SF: Multidimensional Fatigue Symptom Inventory-Short Form, NA: not applicable, N-OB: non-obese, NR: not reported, OB: obese, PCS: physical component score, Post: end/length of intervention, PROMIS: patient-reported outcomes measurement information system, PSQI: Pittsburgh sleep quality index, PSS: perceived stress scale, QLACS: quality of life in adult cancer survivors, RCT: randomised controlled trial, reps: repetitions, SD: standard deviation, SI: sexual interest, SF: sexual function, SF-36: short form 36, USA: United States of America
*denotes significance at *p*<0.05, ^#^denotes estimates
